# Imaging Description of Extragenital Müllerian Adenosarcoma: A Case Report

**DOI:** 10.1055/s-0038-1676110

**Published:** 2018-12-12

**Authors:** Annalisa Mone, Piergiorgio Iannone

**Affiliations:** 1 Department of Radiology, University of Verona, Verona, Italy; 2 Section of Obstetrics and Gynecology, Department of Morphology, Surgery and Experimental Medicine, University of Ferrara, Ferrara, Italy

**Keywords:** extragenital müllerian adenosarcoma, computed tomography, pelvic mass, uterine sarcoma, adenosarcoma mülleriano extragenital, tomografia computadorizada, massa pélvica, sarcoma uterino

## Abstract

Müllerian adenosarcoma is a very rare gynecological disease, comprising 5% of uterine sarcomas. Extragenital localizations are even rarer. We report a very interesting case of a 27-year-old woman complaining of pelvic pain, with a subsequent diagnosis of extragenital Müllerian adenosarcoma. This is the first case reported in the literature with a complete and wide imaging description. Even if rare, Müllerian adenosarcoma should be hypothesized in case of young female patients presenting with suspicious pelvic mass.

## Resumo

O adenosarcoma Mülleriano é uma doença ginecológica muito rara, compreendendo 5% dos sarcomas uterinos. Localizações extragenitais são ainda mais raras. Relatamos um caso muito interessante de uma mulher de 27 anos queixando-se de dor pélvica com diagnóstico subsequente de adenosarcoma Mülleriano extragenital. Este é o primeiro caso relatado na literatura com uma descrição completa e ampla de imagem. Mesmo que raro, o adenosarcoma Mülleriano deve ser hipotetizado no caso de pacientes jovens do sexo feminino com massa pélvica suspeita.

## Introduction

Müllerian adenosarcoma is an uncommon variant of the Müllerian mixed tumor of the uterus.^1^ Uterine adenosarcomas make up 5% of all uterine sarcomas and tend to occur in postmenopausal women but may also be diagnosed in adolescents and young women.^2^ The uterus is the most common site of origin, but, even though rarer, it may also arise in extrauterine locations, including the ovary, vagina, fallopian tube, gastrointestinal tract, bladder and peritoneal sites, such as the pouch of Douglas and the intestinal serosa.^3^ ^4^ ^5^ There are no specific risks or etiologic factors, but extrauterine adenosarcoma has been reported to arise in association with endometriosis.^6^ There are also few cases of adenosarcoma arising in women on tamoxifen, and those with endogenous hyperestrogenism or prior pelvic radiation.^7^ ^8^ ^9^ Adenosarcoma is characterized by a biphasic cellular differentiation, its essential features include benign appearing epithelial component and malignant mesenchymal component.^3^ ^4^ ^10^ ^11^ The epithelial component is usually characterized by endometrioid type; the malignant stromal component is typically low grade.^3^ ^6^ The most common symptom of uterine adenosarcoma is vaginal bleeding; some patients complain of pelvic pain, vaginal discharge or symptoms related to uterine enlargement.^9^ Upon examination, the patients often have a polypoid mass protruding through a dilated cervical os.^6^ Extrauterine adenosarcomas have no specific clinical presentation: abdominal discomfort is the most common symptom.^3^ ^6^ ^12^ Müllerian adenosarcomas have a good prognosis, with the exception of deeply invasive tumors or those with high grade sarcomatous overgrowth; extrauterine adenosarcomas also have a higher risk of recurrence.^9^ Risk factors associated with recurrence and metastases include deep myometrial or lymph-vascular space invasion and sarcomatous overgrowth.^6^ ^10^ We report a very interesting case of extrauterine Müllerian adenosarcoma demonstrated on ultrasound and computer tomography (CT) imaging. According to our knowledge, this is the first case described in the literature of extragenital Müllerian adenosarcoma with full and exhaustive images not associated with known risk factors and/or comorbidities.

## Case Description

**Fig. 1:**
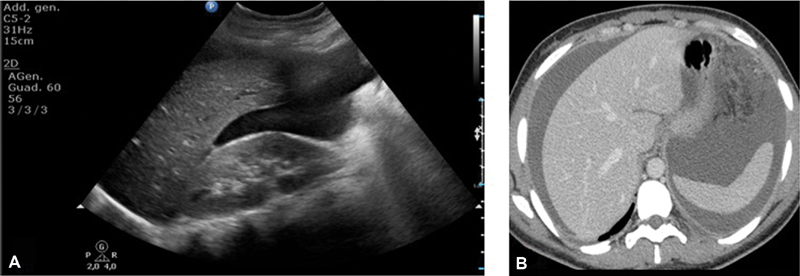
Copious ascites on transabdominal ultrasound (**A**) and CECT (**B**).

**Fig. 2:**
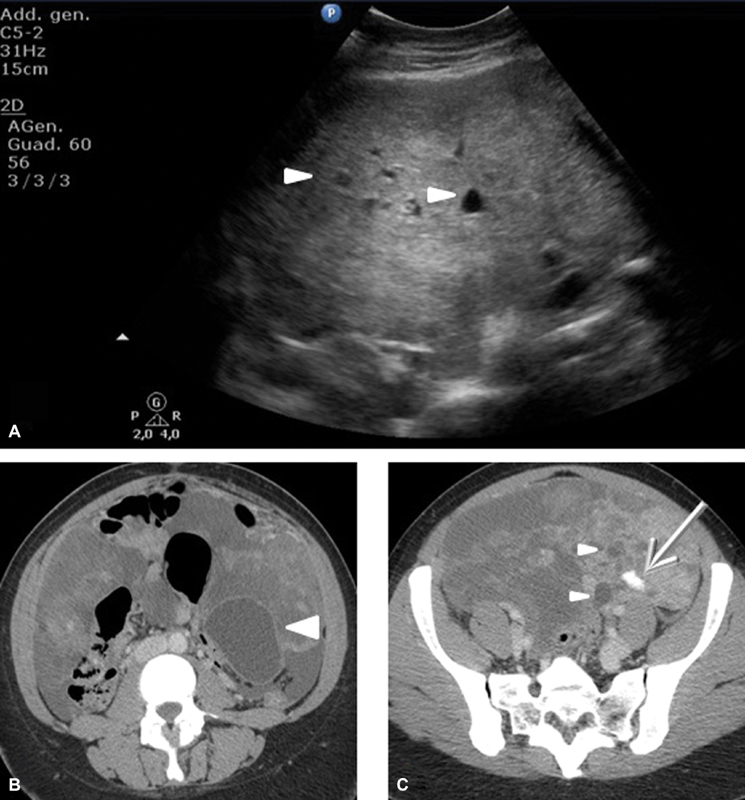
Transabdominal ultrasound (**A**) and axial CECT (**B-C**) images show a large solid, heterogeneous mass containing cystic spaces (▸). Note the presence of calcifications (→) (**C**).

**Fig. 3:**
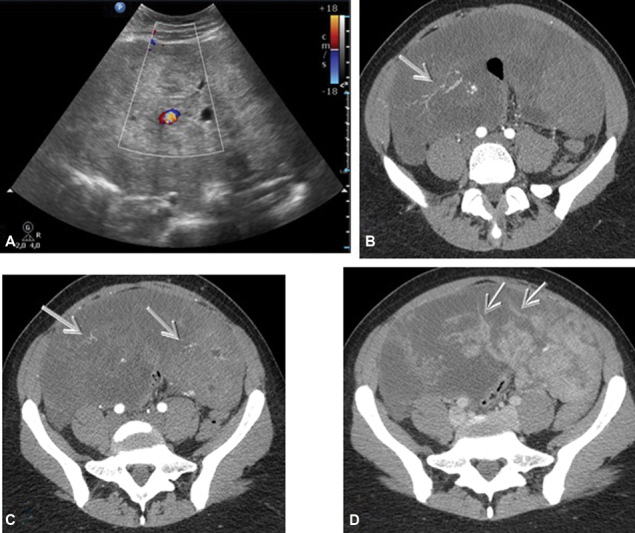
Mass shows internal vascularity on ultrasound color Doppler (**A**) and axial computed tomography during arterial phase (**B-C**) (→). Enhancing on venous/portal phase of septa and pseudosepta (**D**) (→).

A 27-year-old woman refers to our hospital complaining of dyspepsia and abdominal distension for a few months. Her past medical history is silent. Her blood exams show an elevated CA 125 of 1,309 UI/ml. Upon physical examination, she presents considerable abdominal distension and obtuseness to the lower abdominal quadrants as for the presence of ascites. A transabdominal ultrasound, performed using a Philips C5–2 MHz USB curved array transducer (Philips Healthcare, Best, Netherlands) confirms the ascites in all peritoneal recesses (Fig. 1A) and reveals a bulky mass, which occupies almost entirely the abdominal cavity and the pelvis. It appears predominantly solid, with few cystic components and heterogeneous echotexture (Fig. 2A). Internal blood flow is demonstrated on ultrasound color Doppler (Fig. 3A). A 246-CT (Philips Healthcare, Best, Netherlands) of the abdomen and pelvis is performed after a few hours for further evaluation. The protocol used is as follows: precontrast phase and postcontrast tri-phase scan (arterial phase, portal/venous phase and delayed phase). The CT confirms the findings highlighted on the ultrasound: diffused ascites (Fig. 1B) and a multiloculated, voluminous solid mass with definite margins measuring 20 × 10 cm on the axial view, with cranio-caudal extension of 23 cm. The mass occupies partially the abdomen and entirely the pelvis, and presents contextual cystic components, the largest of which was 6 × 4 cm, and calcifications, the largest of which measured 14 mm (Fig. 2B-C). It shows heterogeneous vascularity during the arterial phase (Fig. 3B-C) and presents few septa and pseudosepta, which have a lively enhancement on postcontrast images, especially during the portal/venous phase (Fig. 3D). The mass dislocates the intestinal loops but does not show signs of local invasion. There are no enlarged lymphnodes. The uterus is normal; the ovaries are not viewable. The patient undergoes surgery with the presumed diagnosis of an adnexal cancer. The mass is described as 30 cm in size, with irregular surface and cerebroid appearance. The uterus and the ovaries are normal. Intraoperative pathological consultation reveals borderline tumor of the fallopian tube. Bilateral salpingectomy and total omentectomy are performed. The definitive histological examination shows normal fallopian tubes and Müllerian adenosarcoma arising from the peritoneum. The peritoneal fluid presents no pathological features. The patient is discharged without medical therapy and the hospital stay is uneventful. One month after the surgery, hysteroscopy is performed to obtain endometrial sample, which results negative for neoplasia. Two months after the surgery, the patient undergoes a whole-body CT scan, which shows neither signs of recurrence nor metastasis.

## Discussion

Müllerian adenosarcoma is a very rare tumor and comprises only 5% of uterine sarcomas, occurring in patients from 14 to 89 years old (median 58).^6^ ^9^ The extragenital locations are even rarer, and usually the patients affected by this pathology present a history of endometriosis, tamoxifen therapy, hyperestrogenism or prior pelvic radiation.^9^ Other patients may have a history of recurrent endometrial or cervical polyps; however, these may be coincidental associations without proven etiological factors.^6^ ^9^ In the literature, among the risk factors described, the majority of adenosarcomas are associated with endometriosis.^13^ ^14^ ^15^ ^16^ ^17^ ^18^ ^19^ ^20^ ^21^ ^22^ ^23^ ^24^ Symptoms may vary according to location. In uterine adenosarcoma, the most common presenting symptom is abnormal vaginal bleeding, but some patients present pelvic pain, abdominal mass or vaginal discharge.^9^ In the extragenital locations, abdominal discomfort is the usual presenting symptom.^6^ Histologically, the tumor is characterized by the presence of both epithelial and stromal elements, with the latter predominating.^9^ The epithelial elements usually consist of glands and, in most cases, the epithelium is endometrioid and resembles proliferative endometrium; on the other hand, the malignant stromal component is typical low grade.^6^ ^9^ Mesenchymal elements may also be observed.^9^ Adenosarcomas with more than 25% of the tumor composed of pure high-grade sarcoma are designated as adenosarcomas with sarcomatous overgrowth, and they may be associated with deep myometrial and vascular invasion.^6^

In most adenosarcomas with a low-grade stromal component, the stromal element expresses estrogen receptor, progesterone receptor, CD10, WT1, smooth muscle actin, low MIB1 proliferation index and is negative for P53.^6^ ^9^ The CD10 is diagnostically a useful marker for endometrial stromal tumors and can also be used in establishing the diagnosis of uterine adenosarcomas.^6^ Alterations in the PIK3CA/AKT/PTEN pathway were also found.^9^ Immunohistochemistry of our patient presented high level of estrogen/progesterone receptors, smooth muscle actin positivity and low MIB 1 proliferation index. Outside the uterine cavity, the main locations of the cancer described are the ovary, peritoneum, pelvis and, in patients who underwent hysterectomy, the vagina.^14^ ^17^ ^18^ ^19^ ^20^ ^21^ ^22^ ^23^ ^24^ ^25^ Other sites where extragenital Müllerian adenosarcoma may be found are the rectum and vaginal-rectum septum.^13^ ^15^ ^16^ In 2001, Hirakawa et al^24^ have interestingly reported for the first time the presence of tumor cells in the ascitic fluid in a patient presenting adenosarcoma of the ovary.^24^ In our patient, the peritoneal fluid presented no pathological cells. Only in one case, it was described that a patient with history of endometriosis presented with extragenital adenosarcoma and concomitant colon-rectal neoplasm, but the association between the two tumors has not been studied yet.^15^ Patients with Müllerian adenosarcoma have a generally good prognosis, unless the tumor shows deep myometrial invasion or sarcomatous overgrowth.^9^ Among the cases present in the literature, only a few have been reported with a histologic description of “sarcomatous overgrowth,” a very rare condition with a worse prognosis.^18^ ^21^ ^23^ Recurrences, which occur in 20 to 30% of patients, are usually confined to the vagina, pelvis or abdomen (44% recurrence rate of adenosarcomas with sarcomatous overgrowth compared with 14% without sarcomatous overgrowth).^6^ ^9^ Anyway, long term follow-up is needed.^9^ The treatment of uterine adenosarcoma is hysterectomy with bilateral salpingo-oophorectomy; however, ovarian preservation can be considered in premenopausal women; treatment decisions need to be individualized based on age and clinical/pathological parameters.^6^ This is the first case reported in the literature, with a wide and complete imaging description of this lesion, in a patient with a silent medical history, without known risk factors and/or comorbidities, such as endometriosis. Interestingly, this is the second case reported in Italy.^16^

## Conclusion

Müllerian adenosarcoma should be included in the differential diagnosis of young patients presenting with complex masses in. Although Müllerian adenosarcoma is a very rare disease, more studies are needed to improve the knowledge of this condition to provide its diagnostic, clinical and therapeutic management.
